# Supplementary figures and images for: Multi-locus genotyping reveals established endemicity of a geographically distinct Plasmodium vivax population in Mauritania, West Africa
Source: PLoS Negl Trop Dis. 2020 Dec 16;14(12):e0008945. doi: 10.1371/journal.pntd.0008945 (PMC7773413; doi:10.1371/journal.pntd.0008945)

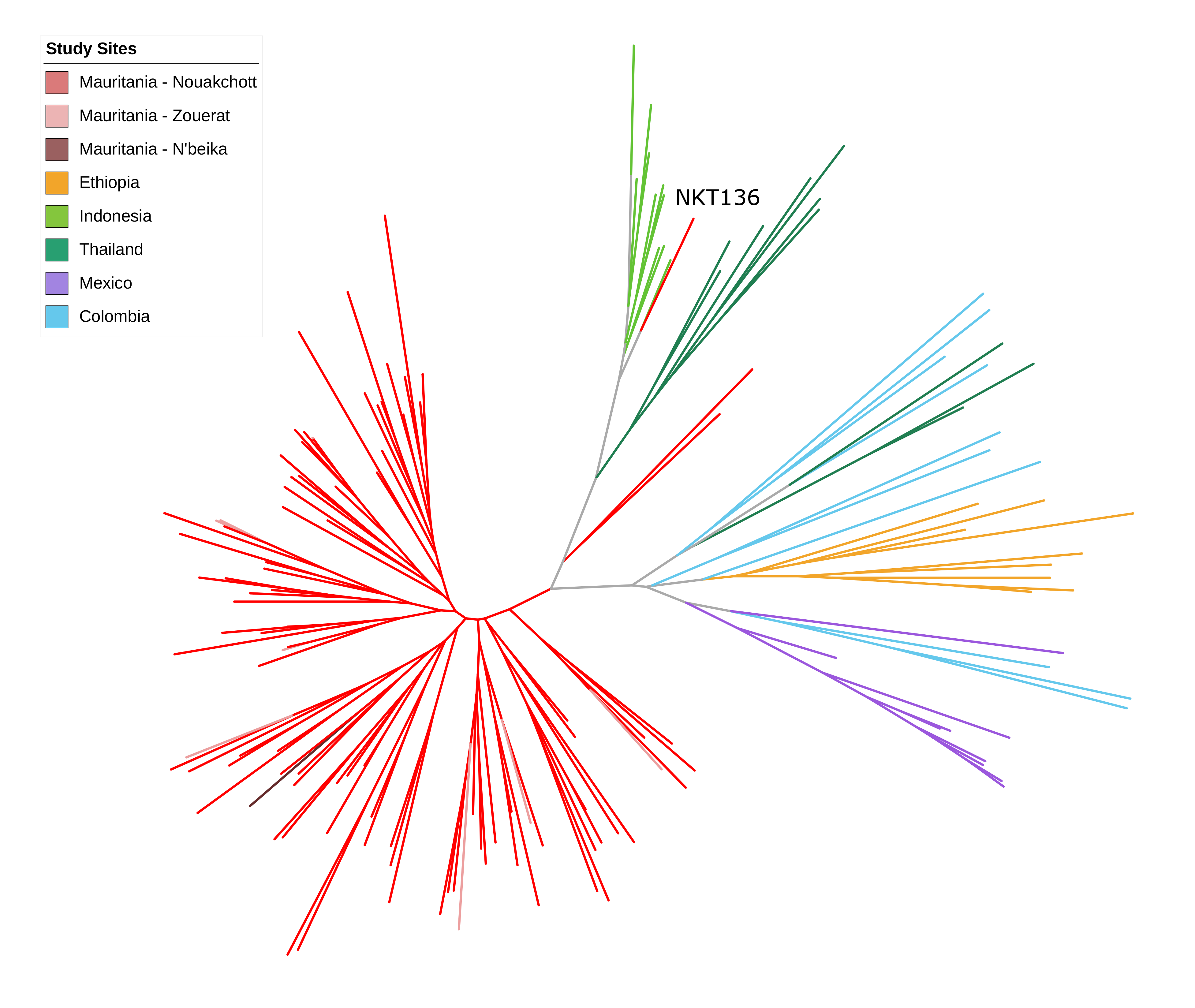

Supplement: S1 Fig — Note that the topology is similar to the rooted tree in Fig 2, and a single isolate from Mauritania (NKT136) has a genotype similar to Southeast Asian parasites. Note that this is not a phylogenetic tree but a genetic distance dendrogram of a recombining species. (TIFF) [file pntd.0008945.s001.tiff]

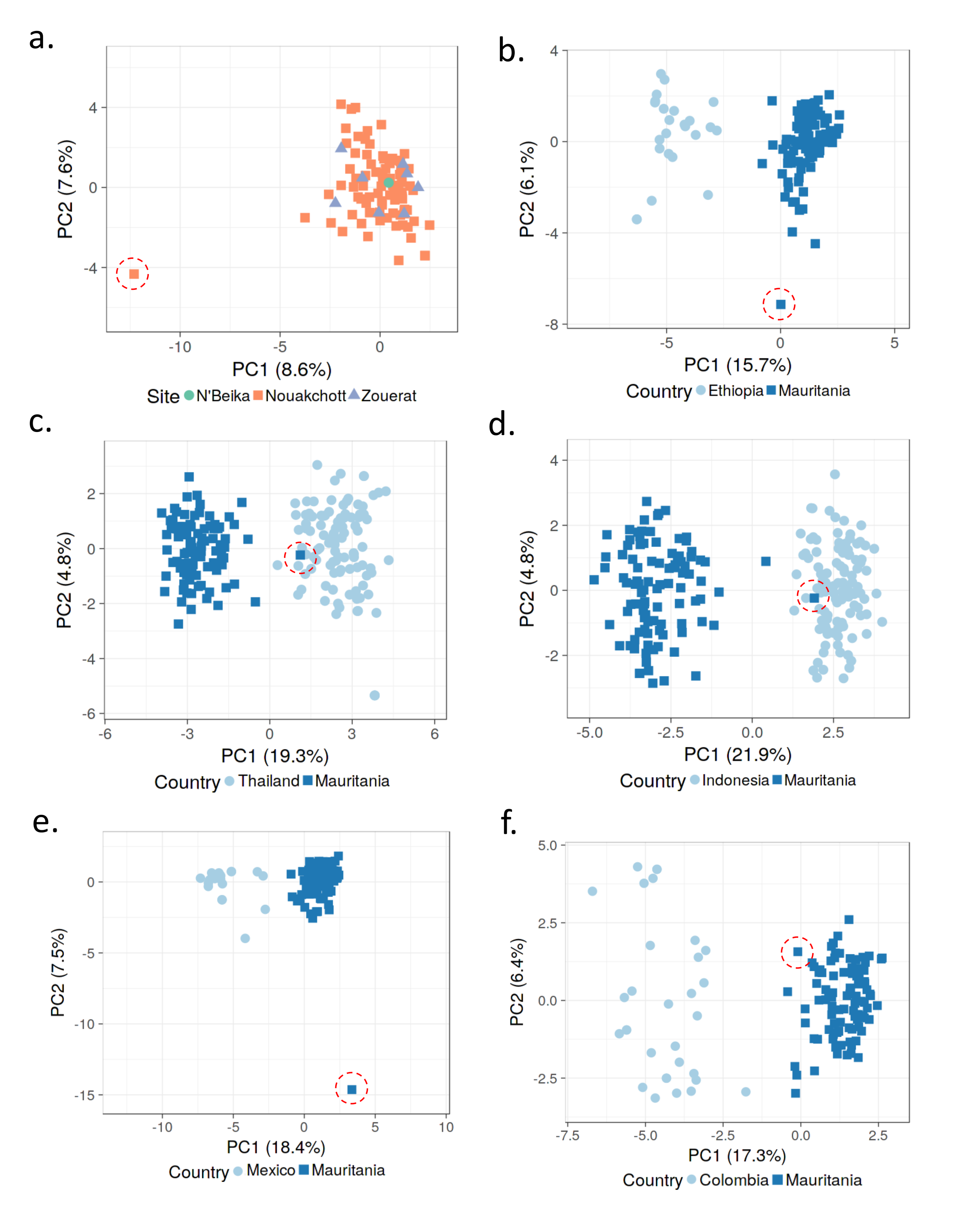

Supplement: S2 Fig — PCA of P. vivax genotypes (38-SNP array) of individual clinical isolates from different pairs of sites, comparing data from Mauritania in this study with data previously published from elsewhere: (a) Within Mauritania (Zouerat, N’beika and Nouakchott), (b) Mauritania and Ethiopia, (c) Mauritania and Thailand, (d) Mauritania and Indonesia, e) Mauritania and Mexico, f) Mauritania and Colombia. This shows no genetic separation of parasites from different sites within Mauritania, but separation of the Mauritanian population from parasites in each of the other countries (previous data from Ethiopia, Thailand, Indonesia, Mexico and Colombia as cited in the Methods and Results). The outlier isolate NKT136 which shows genetic similarity to those from Southeast Asia is circled. (TIFF) [file pntd.0008945.s002.tiff]
