# Supplementary material for: Multi-locus genotyping reveals established endemicity of a geographically distinct Plasmodium vivax population in Mauritania, West Africa
Source: PLoS Negl Trop Dis. 2020 Dec 16;14(12):e0008945. doi: 10.1371/journal.pntd.0008945 (PMC7773413; doi:10.1371/journal.pntd.0008945)
Supplement: S3 Table — (DOCX) [file pntd.0008945.s005.docx]

**Supplementary Table S3.** Genetic differentiation between the Mauritanian *P. vivax* population sampled in this study and previously described populations.

|  | **Mauritania** | **Ethiopia** | **Thailand** | **Indonesia** | **Mexico** | **Colombia** |
| --- | --- | --- | --- | --- | --- | --- |
| **Mauritania** | - | 0.14 | 0.18 | 0.20 | 0.17 | 0.16 |
| **Ethiopia** | 0.22 | - | 0.14 | 0.22 | 0.25 | 0.14 |
| **Thailand** | 0.28 | 0.27 | - | 0.13 | 0.2 | 0.13 |
| **Indonesia** | 0.31 | 0.32 | 0.21 | - | 0.3 | 0.25 |
| **Mexico** | 0.29 | 0.33 | 0.39 | 0.44 | - | 0.11 |
| **Colombia** | 0.23 | 0.2 | 0.21 | 0.33 | 0.17 | - |

Hudson’s *F*_ST_ index is shown in the lower matrix, Weir & Cockerham’s *F*_ST_ index in the upper matrix, each based on the mean of the array of 38 SNPs with data as described in the Methods and Results.
