# Supplementary material for: Multi-locus genotyping reveals established endemicity of a geographically distinct Plasmodium vivax population in Mauritania, West Africa
Source: PLoS Negl Trop Dis. 2020 Dec 16;14(12):e0008945. doi: 10.1371/journal.pntd.0008945 (PMC7773413; doi:10.1371/journal.pntd.0008945)
Supplement: Attachment — Submitted filename: Responses to Reviewers.docx [file pntd.0008945.s007.docx]

We grateful for the well-considered reviews of our manuscript ‘Multi-locus genotyping reveals established endemicity of a geographically distinct *Plasmodium vivax* population in Mauritania, West Africa’, and for your recommendation to make minor revisions in response to the reviewers’ specific comments. We appreciate the appropriate and helpful points made by the reviewers and have revised the manuscript accordingly, as follows (noted in bold italics below):

Methods

Reviewer #1:
Include in the Introduction the treatment scheme for *Plasmodium vivax* in the region and information to support the drug resistance-associated SNP alleles studied. The objectives are well outlined in the face of the problem presented. The methodology described is adequate to achieve the proposed objectives. The sample allows for the proposed investigation. Statistical analyses are adequate.

***We have added the information on treatment to the Introduction as requested.***

Reviewer #2:

The objectives of the study are clearly articulated. The study design is appropriate to the objectives. A few additional details would be welcome:

1. What is the primary method of malaria diagnosis performed in Mauritania (microscopy or RDTs)? If RDTs, is the standard a *Plasmodium falciparum* specific RDT?

***We have added a note on this in the Introduction. At the time of sampling slide microscopy was the primary method, and although use of RDTs has increased recently (recognising undifferentiated Plasmodium species as well as P. falciparum specifically), cases may be still diagnosed presumptively in some routine situations.***
2. It would be recommended to address the human genetic differences in Mauritania (similar to Ethiopia) that partially contribute to the high prevalence relative to other regions in sub-Saharan Africa and also differentiate between infection in Duffy positive individuals in Mauritania in contrast with the accounts of Duffy negative *P. vivax* infection in other SSA countries.

***We have added information on the Duffy positive status of all patients analysed here in the Materials and Methods as we had tested this previously.***

Results

Reviewer #1:

The results are well presented with very informative tables. Improve the map (Fig 1) of Mauritania to locate the area on the respective continent.

***We have modified Fig 1 as suggested.***

Reviewer #2:

The results are clearly and completely presented and the analysis presented is appropriate given the analysis plan. It would be interesting to have more details in the following areas:

• While the individuals in the study reported no travel, more detail on the samples from Zouérat, the mining town would be welcome. Did these samples come from residents or migratory miners? The results indicate that these populations were interspersed with the samples from Nouakchott in terms of genetic diversity, but it would be interesting to have some more details of the underlying patient population from this unique site.

***We have added information in the Materials and Methods clarifying that this is a long-established urban community with most miners not being migrant but having families settled there.***
• Lines 225-227: Both genetic analysis and drug resistance allele indicate a single sample of imported malaria from Asia. Would it be possible to epidemiologically track the origins of this importation or at least to provide more patient metadata that might clarify when this parasite entered Mauritania and how?

***The protocol does not allow for retrospective tracking of behaviour of individual cases, although travel in the two weeks prior to diagnosis was excluded, so the likely prior origin of this single infection is unknown.***
• The authors note that the samples were genetically diverse and only three pairs of infections had identical multi locus genotypes and were not epidemiologically linked. The authors comment on the temporal spacing of the samples with identical multi-locus genotypes, but has fine spatial mapping been performed to determine if identical samples in Nouakchott might show any neighborhood or household clustering patterns?

***Spatial mapping of residences of patients was not performed, as recruitment was at health facilities and the protocol did not include retrospectively focusing on individual cases that showed unusual genotypic patterns.***

Conclusions

Reviewer #1:

The conclusions adequately support the results obtained. I suggest the authors to correlate the data found based on the data observed in the manuscript “The Evolutionary History of *Plasmodium vivax* as Inferred from Mitochondrial Genomes: Parasite Genetic Diversity in the Americas. Taylor et al . Mol Biol Evol. 2013 Sep; 30 (9): 2050–2064”.

***This reference is now cited in the Discussion.***

Reviewer #2: The conclusions are supported by the data.
• It would be interesting if the authors could provide some clinical context on what is the standard procedure for detection and treatment of *P. vivax* in Mauritania and to comment of hypothesize why it has gone so largely undetected and untreated relative to other global populations.

***Information on this has been added in the Introduction and Discussion, noting that the procedures for P. vivax have not been different than for P. falciparum in Mauritania, but that future control will require radical treatment of P. vivax.***
• It would be helpful to have a little more data/clarification for the conclusion that "endemic infection (*P. vivax*) has been more neglected in comparison to *P. falciparum* locally”. Have comparisons by this group or others been specifically performed between Pf and Pv in this population?

***We have modified the wording of this sentence as formal comparisons of levels of neglect have not been made, and we clarify how P. vivax has not been widely recognised or focused on locally except in very recent research studies.***
• Lines 302-310: The authors speculate that given the rare rainfall that it is likely that most of the *P. vivax* infections are from relapses. Given this, what would be the most appropriate strategy for Mauritania to achieve malaria elimination and what would the caveats be with this approach? The paper has clearly and convincingly described the history and nature of population structure in Mauritania; however, a more detailed discussion of how these data can be helpful to advance our understanding and resulting intervention would be helpful for the public health relevance.

***We have added to the Discussion to indicate the need for radical treatment of P. vivax to reduce the reservoir of chronic infections that has not been well appreciated previously, while other approaches to control are also needed that can be considered in the light of the results of this study and others.***

Editorial and Data Presentation Modifications

Reviewer #1: Overall, the manuscript presents relevant information for those working with epidemiology, population genetics and malaria treatment by *Plasmodium vivax*. The study aims to investigate the P. vivax population genetic structure in Mauritania and compare with populations previously analyzed elsewhere, multi-locus genotyping. The work is able to be published in PLOS Neglected Tropical Diseases after minor revisions

Reviewer #2: The only potential modifications might be additional detail on the patient populations and the related epidemiology.

***We have added information relating to this in response to the points above.***

The changes are clearly outlined in a copy of the revised manuscript with ‘track changes’.
